# Complications: Learning From Sharing

**DOI:** 10.1016/j.jaccas.2025.103232

**Published:** 2025-02-05

**Authors:** Andrea Scotti

**Affiliations:** Department of Interventional Cardiology, Montefiore-Einstein Center for Heart and Vascular Care, Montefiore Medical Center, Albert Einstein College of Medicine, Bronx, New York, USA

**Figure:**
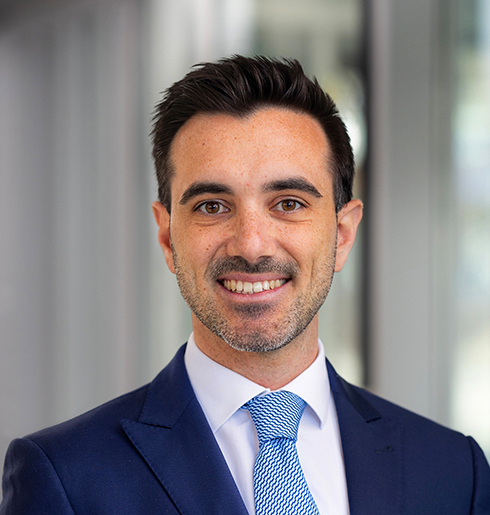

In this ever-evolving landscape of cardiovascular medicine, innovation and expertise have enabled us to tackle some of the most challenging conditions with remarkable success. However, even as procedural techniques and technologies advance, the complexity of cardiovascular interventions often brings with it the potential for complications—some expected, others rare and unanticipated.

This issue of *JACC: Case Reports* is dedicated to exploring these complications, not to dwell on the difficulties, but to emphasize the profound value of shared knowledge in our collective pursuit of excellence. Complex cases and unexpected outcomes are often the best teachers, revealing nuances in patient care that are not readily apparent in clinical trials or procedural guidelines. Each complication is a story, a combination of patient-specific factors, procedural intricacies, and sometimes sheer unpredictability. By openly discussing these challenges, we not only enhance our understanding but also create a repository of insights that can prevent similar occurrences in the future. It is through this open exchange that we elevate the standard of care for our patients (Figure 1).

**Figure 1:**
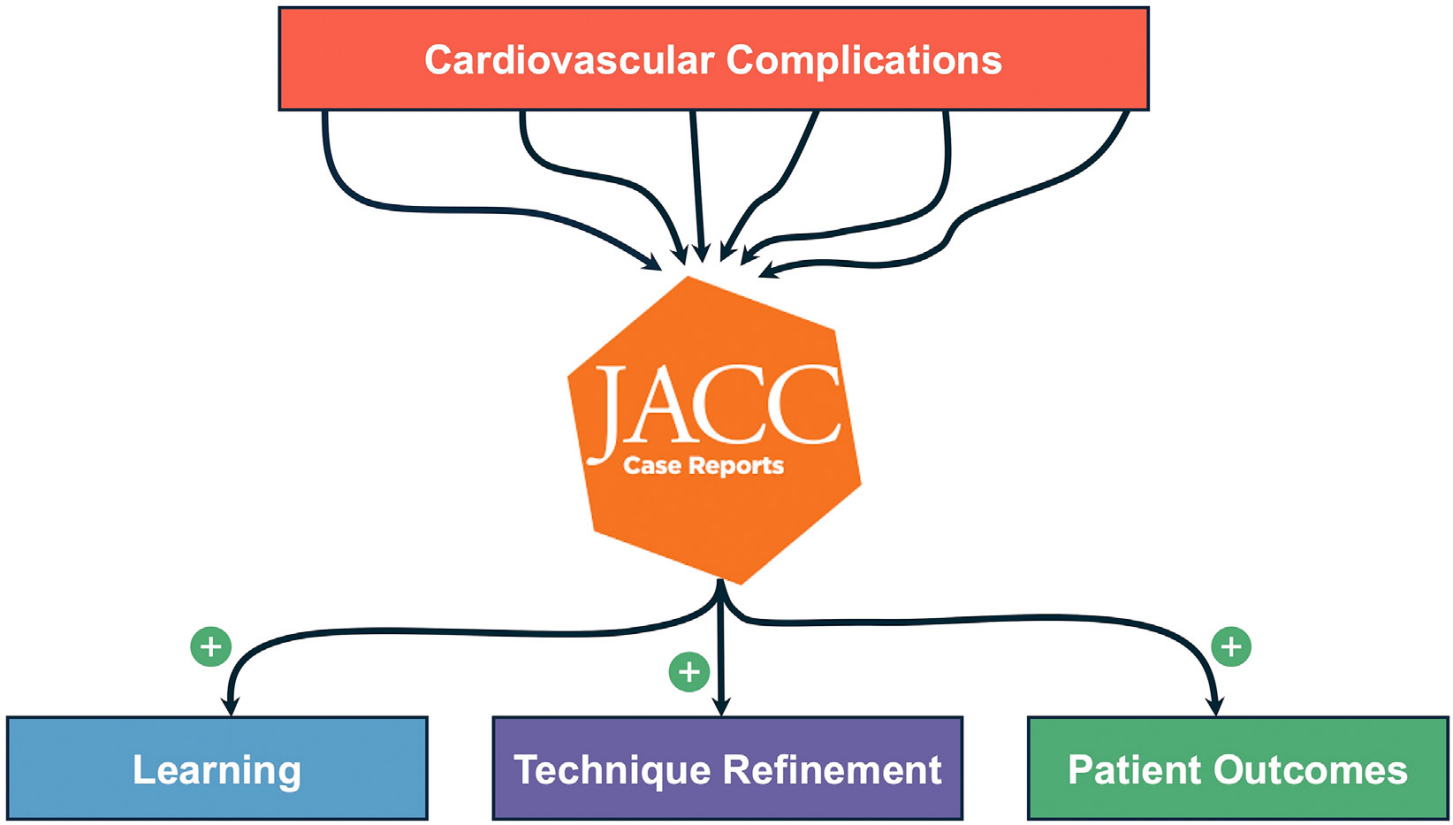
The Value of Learning From Complications in Cardiovascular Medicine

Within these pages, you will find case reports, analyses, and expert commentaries that shed light on complications encountered in various cardiovascular procedures. These include issues such as device malfunctions, anatomical variations, procedural missteps, and unexpected postprocedural outcomes. For every patient whose case presents a hurdle, there are countless clinicians who can learn from it. Each piece offers not only a description of the complication but also reflections on its management and the lessons learned.

Importantly, this issue of *JACC: Case Reports* underscores the ethos of collaboration in cardiovascular care. The willingness to share complex cases and candidly discuss what went wrong is a testament to the dedication of health care professionals to continuous improvement. It is an act of humility and courage, driven by the understanding that shared experiences benefit the entire medical community and, most importantly, our patients.

## Funding Support and Author Disclosures

The author has reported that he has no relationships relevant to the contents of this paper to disclose.

